# Long Term Evaluation of Quantitative Cumulative Irradiation in Patients Suffering from ILDs

**DOI:** 10.3390/diagnostics14192136

**Published:** 2024-09-26

**Authors:** Julien Berg, Anne-Noelle Frix, Monique Henket, Fanny Gester, Marie Winandy, Perrine Canivet, Makon-Sébastien Njock, Marie Thys, Colin Desir, Paul Meunier, Renaud Louis, Francoise Malchair, Julien Guiot

**Affiliations:** 1 Department of Respiratory Medicine, University Hospital of Liège, 4000 Liège, Belgiummarie-laurence.winandy@chuliege.be (M.W.); ms.njock@chuliege.be (M.-S.N.);; 2 Department of Radiology, University Hospital of Liège, 4000 Liège, Belgium; 3 Department of Biostatistics and Medico-Economic Information, University Hospital of Liège, 4000 Liège, Belgium; 4 Department of Clinical Science, University of Liège, 4000 Liège, Belgium

**Keywords:** interstitial lung disease, irradiation, CT scan

## Abstract

Background: Interstitial lung diseases (ILDs) are an heterogeneous group of infiltrating lung pathologies, for which prompt diagnosis and continuous assessment are of paramount importance. While chest CT is an established diagnostic tool for ILDs, there are no formal guidelines on the follow-up regimen, leaving the frequency and modality of follow-up largely at the clinician’s discretion. Methods: The study retrospectively evaluated the indication of chest CT in a cohort of 129 ILD patients selected from the ambulatory care polyclinic at University Hospital of Liège. The aim was to determine whether the imagining acquisition had a true impact on clinical course and follow-up. We accepted three different situations for justifying the indication of the CTs: clinical deterioration, a decrease in pulmonary function tests (at least a 10% drop in a parameter), and monitoring for oncological purposes. The other indications, mainly routine follow-up, were classified as “non-justified”. Radiation dose output was evaluated with Computed Tomography Dose Index (CTDI) and Dose Length Product (DLP). Results: The mean number of CT scans per patient per year was 1.7 ± 0.4, determining irradiation in CTDI (mGy)/year of 34.9 ± 64.9 and DLP in (mGy*cm)/year of 1095 ± 1971. The percentage of justified CT scans was 57 ± 32%, while the scans justified a posteriori were 60 ± 34%. Around 40% of the prescribed monitoring CT scans had no impact on the management of ILD and direct patient care. Conclusions: Our study identifies a trend of overuse in chest CT scans at follow-up (up to 40%), outside those performed for clinical exacerbation or oncological investigation. In the particular case of ILD exacerbation, CT scan value remains high, underlying the benefit of this strategy.

## 1. Introduction

Interstitial lung diseases (ILDs) represent a heterogeneous group of parenchymal diseases of the lungs, with highly variable clinical courses and outcomes [1,2,3,4,5]. The entire pulmonary architecture, including the interstitium and alveoli, can be damaged, leading to an abnormal scarring process and inducing progressive lung fibrosis. The functional assessment of the evolution of ILDs is still challenging and is mainly based on pulmonary function tests (PFTs), and more particularly on lung volume and diffusion capacity decline [2,6]. Indeed, over time, patients suffering from a progressive fibrosing ILD can experience a significant decline in lung function, principally characterized by a reduction in total lung capacity (TLC) and residual volume (RV), sometimes evolving towards a severe restrictive syndrome. Similarly, the forced vital capacity (FVC) and the maximal expiratory volume in one second (FEV1) are also generally reduced, resulting in a global reduction in the lung volumes, without obstructive syndrome. The lung diffusing capacity for carbon monoxide (DLCO) is also reduced, although this does not systematically correlate with the course of the pathology [7]. Similarly, multiple biomarkers have been developed over time to help clinicians in the diagnosis and follow-up of ILDs [8,9,10].

**Figure 1:**
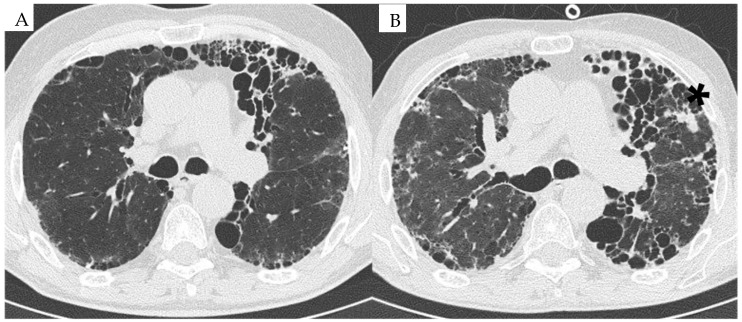
HRCT image of a patient with RA-ILD at baseline in 2015 (**A**) and follow-up in 2023 (**B**). (**A**) UIP pattern associated with RA associated with reticulations and mild ground-glass opacities. (**B**) Progression of the UIP pattern with the occurrence of a neoplastic lung nodule in the left upper lobe (*).

Parallel to the biological and functional evaluation, chest high-resolution CT (HRCT) has a central role in the diagnostic work-up and longitudinal follow-up of patients. Indeed, in addition to assessing the radiological patterns of ILDs, chest CT is of utmost importance in identifying the inherent progression of the disease, or sometimes in highlighting the appearance of concomitant processes, such as neoplastic pathology, which can incidentally be uncovered in some patients, more frequently in these patients with ILD [11]. In HRCT, image acquisition is obtained in a supine position at the end of inspiration, or in some specific cases in sustained expiration. Additionally, the prone position can sometimes be required to reduce any posterior alveolar collapse that can mimic ground-glass opacities. During follow-up, a low irradiating protocol referred to as “low-dose” chest CT can also be used (2 mSv versus 7 mSv for the high-resolution scanner). It is worth nothing that, in the last few years, a new ILD phenotype characterizing patients with a progressive fibrosing interstitial lung disease (other than IPF) has been described by Mouhamad et al. [12], highlighting the undeniable utility of repeated thoracic HRCT evaluation. Of note, contrast utilization is mainly used to rule out pulmonary embolism in cases of unusual or rapidly progressive dyspnea. Therefore, considering the extensive utility of chest CT in ILDs for diagnostic or monitoring purposes, it appears important to unveil the question of the cumulative irradiation of patients engendered by the acquisition of CT images during follow-up (Figure 1), potentially generating long-term effects (stochastic effects). There is no standardized guideline or indication (out of significant clinical worsening) for reassessing chest CT in ILDs, despite the increased risk of neoplastic occurrence [13].

While CT scans are used as a cornerstone for ILD management, their interpretation is prone to large inter- and intra-reader variability, depending on imaging acquisition parameters, the scanner used, and operator experience. The mainstay in drug development for ILD studies has been identified in the change in PFTs and, in particular, FVC, which act as a surrogate for mortality. Also, in the therapeutic follow-up of patients treated with antifibrotic drugs, the use of PFTs is regarded as the standard of care, and the most used surrogate endpoint, which has been correlated with survival [14,15]. A decrease in FVC is associated with an increased risk of mortality, and has been used as primary endpoint for many clinical trials [16,17]. However, chest CT scans are still the preferred choices for ILD patients’ management and therapy planning in current clinical practice, and the issue of radiation exposure and dose accumulation is seldom addressed. To assess the radiation output, some parameters can be referred to, such as the CTDI (an acronym for Computed Tomography Dose Index, measured in mGy), which is a standardized index that measures the radiation dose output applied to a patient per CT section. Another parameter is the dose × length product (measured in mGy*cm), which corresponds to the absorbed dose multiplied by the length explored and is a better reflection of the total dose delivered to the patient. It is important to differentiate measurable physical doses expressed in mGy (amount of energy locally deposited) and non-measurable doses expressed in mSv (quantification of effects and risk assessment). For a chest CT, the conversion factor is 0.017 when converting mGy to mSv [18,19]. The harmful effects of ionizing radiation can be divided into stochastic and deterministic. Stochastic radiation causes effects where the likelihood (but not severity) is directly proportional to the dose. These include malignant tumors and damage to genetic material. These effects occur after a long latency period—from five to twenty years after the exposure. Deterministic radiation causes effects that are dose-dependent in their likelihood and severity, but do not occur below a certain dose level (threshold dose). These effects occur immediately after the exposure to the radiation, most often within 2 to 4 weeks. They are associated with an exposure to high doses above 100 mGy. With regard to the stochastic effects, the concept of a linear, non-threshold probability of their occurrence is adopted, where even the lowest dose of radiation causes a certain risk of cancer. The doses used in diagnostic imaging are typically below 100 mSv and have stochastic effects [20]. It is assumed that the stochastic effects occur randomly and that the risk of their occurrence depends on the type of ionizing radiation, the type of tissue irradiated and the age of the examined person. The stochastic risk is cumulative, increasing with subsequent exposures [21]. On average, the effective dose delivered to patients from a chest CT scan is about 7 mSv, which is equivalent to 2–3 years of background radiation [22]. The estimated number of CT scans that will lead to the development of a cancer varies widely depending on the specific type of CT examination and the patient’s age and sex: as a general indication, it has been reported that a single CT scan may be associated with an increase in the risk of cancer of approximately 1 in 2000 [23].

Our study focused on the indication for performing a follow-up chest CT in ILDs patients, and the potential impact that the control CT had on the clinical course and follow-up, comparing it with the radiation dose experienced by the patient. This is the first study of this kind, to the best of our knowledge, which also considers the real clinical usefulness of the prescribed CT scan, based on real-world data.

## 2. Materials and Methods

### 2.1. Patients Characteristics

We retrospectively selected patients from our ambulatory care polyclinic at University Hospital of Liège. Patients were recruited based on a clinical evaluation between July 2014 and July 2016. The diagnosis of ILD was made according to the international recommendations of the ATS (American Thoracic Association) [1], with an assessment based on pulmonary function test (PFTs), chest HRCT prescribed by a pneumologist or rheumatologist, bronchoalveolar lavage (when available), as well as the clinical history of the patient. All cases had been discussed in the multidisciplinary discussion of interstitial lung diseases, composed of a pulmonologist, a specialist in pulmonary rehabilitation, a rheumatologist, a radiologist, a pathologist, and a specialist in occupational medicine. Patients had a follow-up from their first pulmonology or rheumatology consultation to their last consultation (at the time of the study) or their death. The protocol was approved by the ethics committee of University Hospital of Liège, (Belgian number: B707201422832; ref: 2022/20). All analysis were performed according to the relevant guidelines.

### 2.2. Pulmonary Function Tests

We performed pulmonary function tests in the laboratory at University Hospital of Liège. All spirometry tests were performed using the pneumotachograph. The forced expiratory volume in one second (FEV1) and forced vital capacity (FVC) were measured following the recommendations of the European Respiratory Society (ERS) [24]. The results were expressed in milliliters and percent predicted. The Tiffeneau index, or FEV1/FVC, was expressed in percent. The total lung capacity (TLC) was measured by body plethysmography according to ERS recommendations. The DLCO and the DLCO/AV ratio were measured by the single-breath carbon monoxide gas transfer method and expressed as percent predicted.

### 2.3. Image Acquisition

We used four different CT scanners to evaluate the standard clinical workflow. The different models were Somatom Egde+ (Siemens Healthineers (Forchheim, Germany)), Emotion 16 (Siemens Healthineers), Revolution (GE HealthCare, Waukesha, WI, USA), and BrightSpeed (GE HealthCare). All the chest CT scans were acquired in the inspiration and supine positions. The majority of the scans were high-resolution CT (HRCT), with a slice thickness of ≤ 1 mm. Also, low-dose CT scans with or without contrast were present for some patients.

### 2.4. Dosimetry Evaluation

Radiation data were collected from the report generated by the CT scanner, displaying the *Computed Tomography Dose Index* (CDTI, in mGy) and *Dose Length Product* (DLP, in Gy*cm). Radiological data were collected for each patient, including the number of total chest CTs performed for the follow-up of ILD. We classified the examinations according to the justification for the acquisition of chest CTs, either as an emergency or routine evaluation. We collected the total administrated irradiation in CDTI and DLP. After an individual de-archiving work, the dosimetry data were extracted from each scanner, collected, and added up individually.

### 2.5. Evaluation of Thoracic HRCT Indication

To evaluate the validity of CT indication for each scan, we separated all the scans performed into two groups: justified indication or non-justified indication. Our judgment was based on the motivation written on the medical request by the prescriber. We specifically looked at 3 different situations for justifying the indication:

- Clinical deterioration (fever, desaturation);
- Significant decrease in PFTs (at least 10% drop in FVC);
- Monitoring for oncological purposes.

We then analyzed the results of the chest CTs to see whether it had impacted the treatment or outcome for the patient, independent of the indication. The CT scans performed for routine follow-up without any other criteria were marked as “not justified”. For those examinations, we also checked whether new data collected after the completion of the scanner could have somewhat changed the management of the patient (e.g., the incidental discovery of a lung nodule, ILD progression requiring a therapeutic modification), redefining these cases as justified a posteriori (that had, in fact, a true impact on management and was therefore truly justified) (Table 1).

To evaluate the impact of follow-up CT more accurately, we specifically analyzed a sub-group of ILD patients who experienced a significant functional reduction (from 10% to 15% in PFT parameters) and searched for correlation with CT indication or cumulative radiation.

### 2.6. Statistical Analysis

The Kolomoronov–Smirnov test is used to test the normality of quantitative data. The results of the comparative analysis are obtained from the student’s unpaired T-test when the data are parametric, and from the Mann–Whitney test when the distribution is not normal. These tests has been performed to compare demographics and clinical data between different groups (divided based on the PFT test results) and to assess differences in radiation dose exposure and clinical justification for the prescription of the chest CT scan. The results are considered significant at the 5% uncertainty level (*p* < 0.05). Calculations are performed using TIBCO Statistica^®^ 13.5.0 software. Another part of the statistical study was carried out using Prism and Statistica software v13.2.

## 3. Results

### Patients’ Characteristics

Patients’ characteristics are listed in Table 2. The cohort consisted of 129 patients. The mean age was 63.7 ± 2.3, with a male predominance (61.2%), and an active smoker predominance (58.9%). The main diagnoses were idiopathic nonspecific interstitial pneumonia (NSIP) 30%, idiopathic pulmonary fibrosis (IPF) 26%, connective tissue disease–pulmonary fibrosis (CTD-PF) 31%, sarcoidosis 18%, cryptogenic organizing pneumonia (COP) 5% and respiratory bronchiolitis interstitial lung disease (RBILD) 3%. The cohort of examined patients represents the current clinical practice, presenting different variants of pulmonary conditions, with a large number of male and active smoker patients.

The pulmonary function tests, as expected, globally identified a restrictive syndrome (initial TLC at 77% predicted) with a marked alteration in diffusion (DLCO 54% at baseline) in the majority of cases. The mean follow-up duration was 3.7 years. By the end of the monitoring, 7% of patients had developed lung cancer (and two hematologic cancers) and 32.6% (*n* = 42) died.

The mean number of CT scans performed per patient was 2.6 scans over the first year of follow-up. Thereafter, patients had approximately 1 CT scan per year, reaching a mean sum of 4.5 CT scans after three years of follow-up and 6.4 CT scans on the overall follow-up (Table 3). The irradiation dose is significant, with a CDTI at 27.3 ± 20.2, 54.5 ± 64.9, and 67.8 ± 52.2 (mGy) and a dose-length product of 897 (±595), 1582 (±908), 2192 (±1474) (mGy*cm) respectively, after 1 year, 3 years, and at the end of the total average care. Globally, the calculated mean irradiation was 34.9 mGy and 1095 mGy*cm per patient per year.

The percentage of justified CT scans was 57 ± 32%, while the justified a posteriori scans were 60 ± 34%. This leaves around 40% of non-justified scans on the overall follow-up time. Considering the patients’ cohorts, this is a sensible number of chest CT scans that were not strictly necessary to the patients’ follow-up and did not find a posteriori justification, either for disease exacerbation or oncological follow-up.

Further analysis was carried out by classifying patients into sub-populations, according to the lung disease progression based on PFTs. We separated patients with stable PFTs and patients with a drop of at least 10% of the absolute value in one PFT parameter (FVC or FEV1) or a drop equal or higher than 15% for DLCO (See Table 4 and Table S1). Our findings highlighted a relationship (*p* < 0.05) between the patients presenting a deterioration in lung function parameters such as FEV1 and FVC and the percentage of a posteriori justified CT scans, whereas the correlation with justification for a priori CT scans did not reach statistical significance. This correlation is not present in the drop in DLCO, except for the DLP. PFTs’ decline (assessed by FVC, FEV1, or DLCO) was not associated with a significant increase in total DLP. Interestingly, this progressive subpopulation did not benefit from more emergency, contrast-enhanced, or low-dose CT scans than patients without a progressive disease. Considering the entire sample (*n* = 820 CT scans), we noticed a low prescription rate in low-dose CT scans (68 CT scans = 8%) or emergency CT scans (68 CT scans = 8%), versus a slightly larger number of contrast CTs (138 CT scans = 17%).

## 4. Discussion

To the best of our knowledge, there is no dedicated longitudinal observational study on the quantification and justification of radiation doses in patients with ILDs. Our study demonstrated that patients did not experience a harmful over-irradiation over time. Nevertheless, we noticed that the overall justification of the chest CT scans was not always sound, except for those linked to acute clinical deterioration, which is in accordance with guidelines’ recommendations in the event of an ILD exacerbation [25]. The composition of our patient’s cohort is in line with what is reported in the literature for a chronic ILD populations, with an increased prevalence of CTD-PF due to the bias induced by the recruitment in our hospital, as a tertiary center. The demographic characteristics are as usually seen in patients suffering from ILD [26,27]. In our study, clinicians performed an average of 1.7 scans/year/patient. We observed a concordance between the a priori indication and the a posteriori validity of the scanners. The approach showed that, overall, 56.9% of scans were justified a priori, whereas 60.15% of them were justified a posteriori. We defined the unjustified CT scans as performed systematically without clear clinical indication or expected result, resulting in no specific clinical or therapeutic response. Therefore, a total of 40% of the performed CT scans were not justified in this patient population.

The ERS guidelines define an ILD exacerbation based on a drop in FVC or DLCO or the need for oxygen support [25]. Therefore, PFTs and their degradation over time, such as a drop of 10% or more in FEV1 or FVC, represent a useful monitoring tool that allows for the study of the evolution of ILDs, and could lead to a therapeutic implication for patients. Based on the ERS recommendations defining significant deterioration in DLCO and FVC, we tried to correlate the number of CT scans performed with the drop in FVC and DLCO over time. Interestingly, no specific correlation between those parameters and the number of CT scans have been identified, neither for FVC or DLCO. The absence of a correlation is possibly due to the low number of acute exacerbations that will only slightly increase the number of CT scans [28].

From the current analysis, it emerges that a significant number of CT scans are prescribed without any diagnostic and therapeutic consequences, suggesting that clinicians are performing some of those CT scans in a procedural way, regardless of the result of the PFTs’ modification over time. Moreover, only a small proportion of the CT scans were low-dose CTs. While it seems obvious to explore lung parenchyma through HRCT during the initial evaluation and diagnosis work-up, it is reasonable to assume that systematic imaging during follow-up could be achieved with low-dose acquisition CTs, particularly for oncological screening purposes [12]. While CT scans remains the best tool to assess oncological malignances and severe pathologies of the lungs [29,30,31], considering low-dose CT scans for basic monitoring could imply a reduction in the irradiation of our patients. In our study, it appears that few CT scans were performed in an emergency setting (8%), which is in line with the low prevalence of acute exacerbation of ILDs corroborated in the literature (4–20%) [32].

In clinical practice, PFTs and clinical evaluation can serve as a sufficient routine follow-up methodology to monitor the evolution of the disease. Therefore, global irradiation could be reduced by individuating the imaging acquisition modalities best suited to each patient and situation. Looking at the radiation dose, it is important to consider that the average irradiation level was 34.9 mGy and 1095 mGy*cm per year and per patient (18.6 mSv), which remains high enough to induce stochastic effects after a few years. Several novel CT techniques have been developed in recent years, like photon-counting detector computed tomography (PCD-CT) or wide-area detector row CT; with these techniques it is possible to reduce radiation dose from 21% to 66%, while maintaining good diagnostic quality of the images [33,34,35]. Notwithstanding this, the majority of not-routine CT scans (i.e., emergency CTs or CTs following clinical or functional deterioration) are justified on a clinical basis, highlighting the absolute usefulness of chest CTs in this precise context. Moreover, ILD patients’ follow-up through CT imaging must be put into perspective with lung cancer screening. Systematic lung cancer screening is not specifically recommended in ILD patients, and is committed to personalized and individualized workup. More specifically, ILD patients exhibit an increased risk of developing lung cancer (smoking history, scarring process, the use of immunosuppressive agents). The incidence of lung cancer in the general population is 100/100,000 inhabitants (i.e., 1%/year) [36]. In smokers, the risk of having lung cancer is 10 to 30 times higher than in a non-smoker population [37]. In a review of the literature, the percentage of lung cancer in patients suffering from ILD is 9.8% to 20% depending on the time of follow-up, with the risk globally increasing over time [38]. In our study, the incidence of lung cancer (7%) is similar to what is seen in the literature, with a median follow-up of 3.7 years [39].

At the best of our knowledge, the long-term correlation between radiation dose and the insurgence of lung cancer in ILD patients has not be explored so far in the literature; thus, it is not possible to compare the results in this patient’s cohort with previous data. Other similar studies were performed to assess radiation dose and cancer risk with the use of CT scan. MirDerikvand et al. [22] reported on the comparison between thoracic diagnostic CT and radiotherapy treatment-planning CT scans. The authors concluded that cancer incidence and mortality risks for radiotherapy-planning CT were higher than for diagnostic CT scans, up to 1.5 times higher in the thoracic area. In another study, Kędzierski and coworkers [21] reviewed a large body of clinical data coming from different clinical registries and trials on the radiation doses in CT examinations of the coronary arteries. Their review highlighted a decrease over time in the radiation doses used for these examination, thanks to technological innovation in the scanner equipment and scanning protocols. The risk of developing cancer related to CT radiation exposure is higher in the lungs and breasts: younger patients have a higher risk of developing lymphoma and breast cancer, while older patients have a higher risk of developing lung cancer. It is also worth noting that the usually effective doses in coronary CT scans are higher (16–26 mSv) than for standard diagnostic CT scans. While several studies in the literature reported in general on the risk of developing cancer associated with CT scans [40,41,42], there are no specific studies tailored to ILD patients and their long-term follow-up. Such a study would be welcomed, but would require a larger population and also considering other possible organs that can develop malignancies, such as breast cancers.

### Limitations

Our study faces some limitations. It was a monocentric retrospective study, and the population was relatively small. PFTs and the measurements of the various parameters (FEV1, FVC, TLC, DLCO, KCO) and their deterioration over time occurred between the start and the end of treatment. Some patients could have experienced initial severe deteriorations modified with specifically dedicated therapy, thus altering disease behavior.

## 5. Conclusions

Thoracic imaging using chest CT is the cornerstone in the evaluation of chronic fibrotic lung diseases. Our retrospective study highlighted that routine follow-up HRCT did not always demonstrate an undeniable clinical utility. The cumulative irradiation is still acceptable, but could be reduced with a more cautious use of chest CT scans, leveraging PFTs and clinical examination for a routine follow-up of disease progression. The occurrence of lung cancer is in line with what has been reported in the past for the ILD population. Further prospective studies will have to demonstrate the potential value of HRCT in lung cancer screening in ILDs to guide clinicians in their daily practice. Moreover, it is highly necessary to combine dedicated AI-based automatized tools with clinical and PFT parameters to increase the accuracy of fibrosis quantification. 

## Figures and Tables

**Table 1:** Evaluation of thoracic CT indication.

Justified Chest CT Scans	Non-Justified Chest CT Scans	
- Clinical deterioration - Decrease in PFTs > 10% - Oncological screening	Routine follow up	
	*Justified* a posteriori	*Non-justified* a posteriori
	Incidental findings (e.g., lung nodule)	No incidental findings

**Table 2:** Patients’ demographics and functional respiratory parameters.

	*n* = 129
**Male *n* (%)**	79 (61.2%)
**Mean Age (**±**SD)**	63.7 (±2.3)
**Active smokers *n* (%)**	76 (58.9%)
**Mean follow-up in years (**±**SD)**	3.7 (±2.3)
**FVC** ± SD (% predicted)	81 (±19)
**FEV1** ± SD (% predicted)	81(±20)
**DLCO** ± SD (% predicted)	54 (±20)
**KCO** ± SD (% predicted)	82 (±25)
**TLC** ± SD (% predicted)	77 (±17)

PFT: pulmonary function test; VC: vital capacity; FEV1: forced expiratory volume; DLCO: diffusing lung capacity for carbon monoxide; KCO: transfer coefficient for carbon monoxide for the lung; SD: standard deviation.

**Table 3:** Data concerning the number of CT scans performed and their irradiation consequences.

**Number of CT/patient**	6.34 ± 4.12
**Low-dose CT/patient**	0.5 ± 1
% Low-dose CT/patient	8 ± 18.5
**Contrast CT scan/patient**	1 ± 2
% Contrast CT scan/patient	17 ± 23
**Emergency CT scan/patient**	0.5 ± 1
% Emergency CT scan/patient	6.7 ± 31
**% Justified emergency CT scan**	94.2
**Number of routine CT scan**	5.6 ± 3.5
% Justification a priori of routine CT scan	57 ± 32
% Justification a posteriori of routine CT scan	60 ± 34
**Number of CT scans**	
1 year	2.6 ± 1.5
3 years	4.5 ± 2.5
On the overall follow-up	6.4 ± 4.1
**Number of mean CT scans/patient/year**	1.7 ± 0.4
**Irradiation CDTI (mGy)/patient**	
1 year	27.3 ± 20.2
3 years	54.5 ± 64.9
On the overall follow-up	67.8 ± 52.2
Irradiation DLP (mGy*cm)/patient	
1 year	897 ± 595
3 years	1582 ± 908
On the overall follow-up	2192 ± 1474
**Irradiation in CDTI (mGy)/year**	34.9 ± 64.9
Irradiation in DLP (mGy*cm)/year	1095 ± 1971

Computed Tomography Dose Index (CDTI, in mGy), Dose Length Product (DLP, in Gy*cm). All values are expressed with mean ± SD.

**Table 4:** Comparison between different parameters and the drop of DLCO into two populations: a drop of 15% and more, and a drop of <15%.

	*Drop of DLCO < 15%*	*Drop of DLCO ≥ 15%*	
	*Median (IQR)*	*Median (IQR)*	*p Values*
*Age*	61 (52–71)	66 (53–75)	0.127779
*Follow up (month)*	44 (22–60)	49 (38–71)	0.040146
*Contrast CT scan*	0 (0–1)	1 (0–2)	0.009261
*% justification* a priori *of emergency CT scan ^1^*	100 (100–100)	100 (100–100)	0.704275
*% justification* a priori *of routine CT scan*	50 (30–71)	50 (33–80)	0.600688
*% justification* a posteriori *of routine CT scan*	50 (33–80)	50 (33–80)	0.978767
*irradiation over 1 year CTDI (mGy)*	28 (16–40)	26 (17–39)	0.676711
irradiation over 1 year PDL (mGy*cm)	793 (394–1111)	700 (554–1174)	0.742729
*irradiation over 3 years CTDI (mGy)*	42 (27–65)	58 (39–75)	0.039473
irradiation over 3 years DLP (mGy*cm)	1352 (973–2204)	1585 (1254–2466)	0.057749
*Total irradiation CDTI (mGy)*	56 (30–85)	78 (47–103)	0.004655
Total irradiation DLP (mGy*cm)	1840 (1050–2641)	2431 (1613–3266)	0.021919
*irradiation/year CDTI (mGy/year)*	17 (10–29)	18 (12–24)	0.815285
Irradiation/year (mGy*cm/year)	692 ± 536	831 ± 799	0.787854
*% low-dose CT scan*	0 (0–14)	0 (0–6.7)	0.236869
*% contrast CT Scan*	0 (0–20)	17 (0–33)	0.031569
*% emergency CT scan*	0 (0–11)	0 (0–14)	0.360847

CDTI: Computed Tomography Dose Index CDTI; DLP: Dose Length Product; IQR: interquartile range; data are analyzed with Mann–Whitney test. ^1^ *n* = 18 for DLCO ≥ 15%, *n* = 16 for DLCO ≤ 15%

## Data Availability

The datasets used and/or analyzed during the current study are available from the corresponding author on reasonable request.

## Supplementary Materials

The following supporting information can be downloaded at: https://www.mdpi.com/article/10.3390/diagnostics14192136/s1, Table S1: Comparison between the drop of different parameters of the pulmonary function test and a parameter of the population or a radiologic parameter.
