# Supplementary material for: Long Term Evaluation of Quantitative Cumulative Irradiation in Patients Suffering from ILDs
Source: Diagnostics (Basel). 2024 Sep 26;14(19):2136. doi: 10.3390/diagnostics14192136 (PMC11476226; doi:10.3390/diagnostics14192136)
Supplement: Supplementary file 1 [file diagnostics-14-02136-s001.zip › diagnostics-3076368-supplementary.pdf]

Long term evaluation of quantitative cumulative irradiation in patients suffering from ILDs

Julien Berg[1], Anne-Noelle Frix[1], Monique Henket[1], Fanny Gester[1], Marie Winandy[1], Perrine Canivet[2], Makon-Sébastien Njock[1], Marie Thys[2], Colin Desir[3], Paul Meunier[3], Renard Louis[1], Francoise Malchair[4], Julien Guiot[1*]

1. Department of Respiratory Medicine, University Hospital of Liège, Liège, Belgium
2. Department of Biostatistics and Medico-Economic Information, University Hospital of Liège, Liège, Belgium
3. Department of Radiology, University Hospital of Liège, Liège, Belgium
4. University of Liège, Liège, Belgium

***Corresponding authors:**

Dr. Julien GUIOT, MD, Ph.D. J.Guiot@chuliege.be

University Hospital of Liège

Avenue de l'Hôpital, 1 4000 Liège

Belgium

Table S1. Comparison between different parameters and the drop of DLCO into 2 populations : drop of 15% and more and drop of <15%.

	Drop of DLCO ≤ 15%	*Drop of DLCO≥15%*	
	median (IQR)	*median (IQR)*	*P values*
Age	61 (52-71)	66 (53-75)	0.127779
Follow up (month)	44 (22-60)	49 (38-71)	0.040146
Contrast CT scan	0 (0-1)	1 (0-2)	0.009261
%justification a priori of emergency CT scan[1]	100 (100-100)	100 (100-100)	0.704275
%justification a priori of routine CT scan	50 (30-71)	50 (33-80)	0.600688
%justification a posteriori of routine CT scan	50 (33-80)	50 (33-80)	0.978767
irradiation over 1-year CTDI (mGy)	28 (16-40)	26 (17-39)	0.676711
*irradiation over 1-year PDL (mGy*cm)*	793 (394-1111)	700 (554-1174)	0.742729
irradiation over 3 years CTDI (mGy)	42 (27-65)	58 (39-75)	0.039473
*irradiation over 3 years DLP (mGy*cm)*	1352 (973-2204)	1585 (1254-2466)	0.057749
Total irradiation CDTI (mGy)	56 (30-85)	78 (47-103)	0.004655
*Total irradiation DLP (mGy*cm)*	1840 (1050-2641)	2431 (1613-3266)	0.021919
irradiation/year CDTI (mGy/year)	17 (10-29)	18 (12-24)	0.815285
*Irradiation/year (mGy*cm/year)*	692 ± 536	831 ± 799	0.787854
% low dose CT scan	0 (0-14)	0 (0-6.7)	0.236869
% contrast CT Scan	0 (0-20)	17 (0-33)	0.031569
% emergency CT scan	0 (0-11)	0 (0-14)	0.360847

CDTI: Computed Tomography Dose Index; DLP: Dose Length Product; IQR: interquartile range; Data are analyzed with Mann Whitney test

[1] n=18 for DLCO ≥ 15% , n = 16 for DLCO ≤ 15 %
